# Supplementary material for: Synergy between c-di-GMP and Quorum-Sensing Signaling in Vibrio cholerae Biofilm Morphogenesis
Source: J Bacteriol. 2022 Sep 26;204(10):e00249-22. doi: 10.1128/jb.00249-22 (PMC9578409; doi:10.1128/jb.00249-22)
Supplement: Supplemental file 1 — Fig. S1 to S6 and Tables S1 and S2. Download jb.00249-22-s0001.pdf, PDF file, 1.2 MB [file jb.00249-22-s0001.pdf]

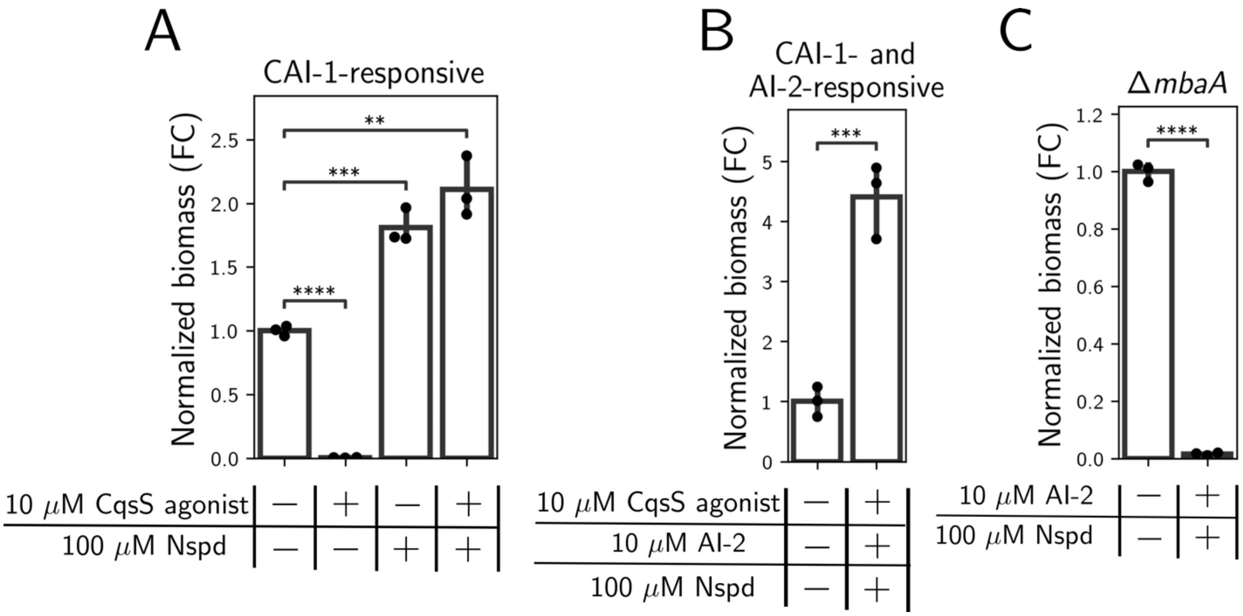


Fig. S1. **Synergy between norspermidine and AI-2 treatments is mediated by the NspS-MbaA and quorum-sensing pathways.** (A) Peak biofilm biomass, measured by quantitative brightfield imaging of biofilms produced by the CAI-1-responsive strain grown with the indicated treatments. (B) Peak biofilm biomass produced by the CAI-1- and AI-2-responsive strain grown with the indicated treatments. (C) Peak biofilm biomass produced by the Δ*mbaA* AI-2-responsive strain, grown with the indicated treatments. Data are normalized as fold changes relative to the untreated strains. *N* = 3 biological replicates. Unpaired *t*-tests were performed for statistical analyses. *****P* ≤ 0.0001; ****P* ≤ 0.001; ***P* ≤ 0.01. Norspermidine, Nspd; Fold change, FC.

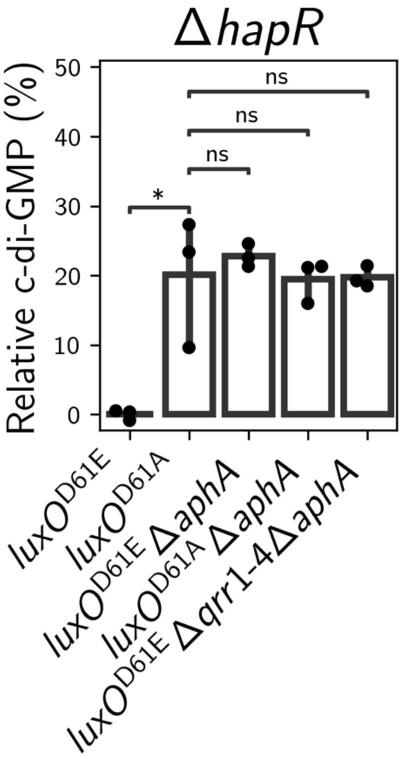


Fig. S2. **AphA activity lowers cytoplasmic c-di-GMP levels.** c-di-GMP reporter output in the indicated *V. cholerae* strains. Data are normalized as percent changes relative to the c-di-GMP produced by the *luxO*$^{D61E}$ Δ*hapR* strain. The LuxO$^{D61E}$ mutant protein mimics LuxO~P, thus locking *V. cholerae* in the low cell density quorum-sensing state. The LuxO$^{D61A}$ mutant protein is incapable of phosphorylation, thus locking *V. cholerae* in the high cell density quorum-sensing state. Unpaired *t*-tests were performed for statistical analyses. *$P \leq 0.05$; ns $P > 0.05$.

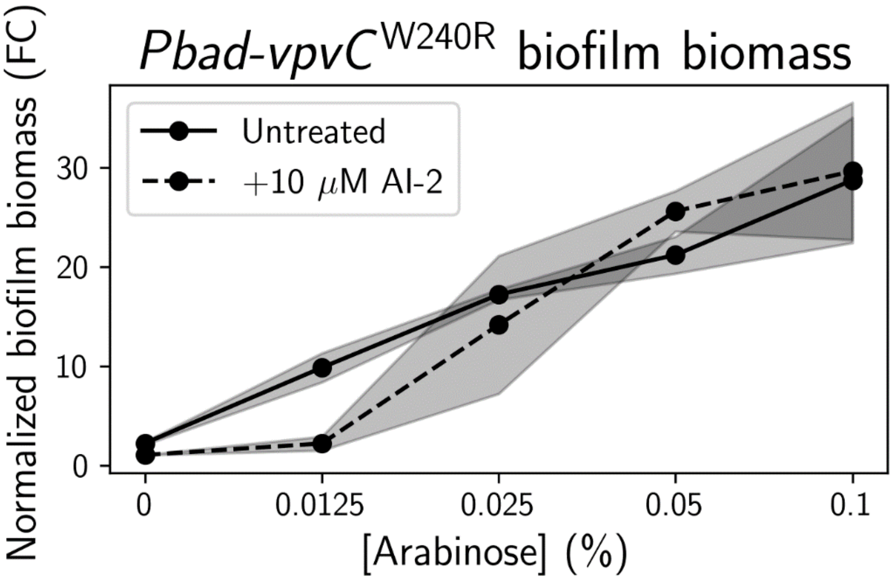


Fig. S3. **Quorum sensing does not generally alter the sensitivity of biofilms to changes in cytoplasmic c-di-GMP levels.** Peak biofilm biomass in the AI-2-responsive strain carrying chromosomal *Pbad*-*vpvC*$^{W240R}$, grown with the indicated treatments. Data are normalized as fold changes relative to the untreated strain at 0% arabinose.

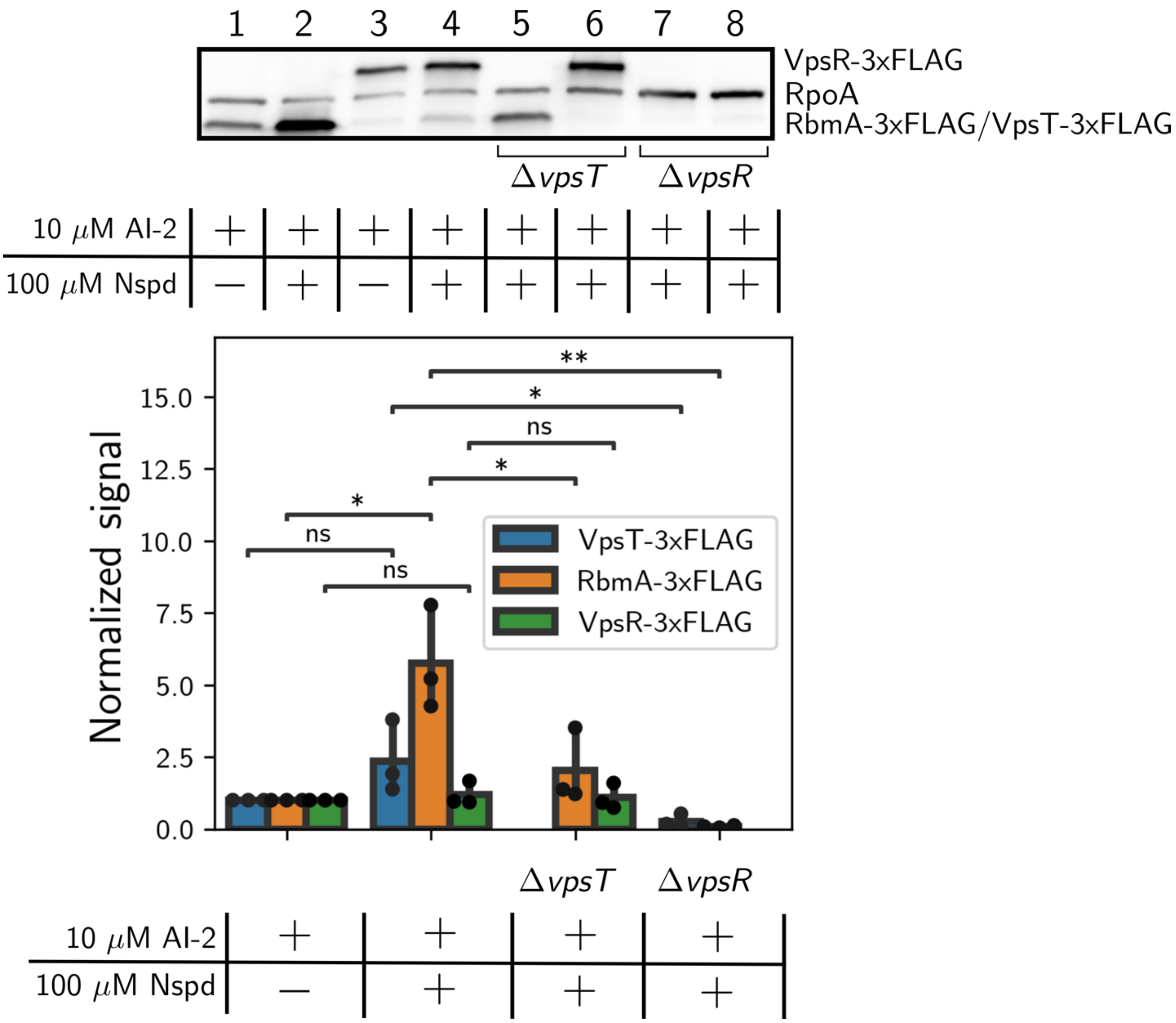


Fig. S4. **VpsR activates *vpsT* and *rbmA* expression in the high cell density and high norspermidine signaling regime.** Top: representative western blot of the indicated proteins in *V. cholerae* grown with the indicated treatments. Lanes 1-4 show the AI-2-responsive strain, lanes 5 and 6 show the Δ*vpsT* AI-2-responsive strain, and lanes 7 and 8 show the Δ*vpsR* AI-2-responsive strain. RbmA-3xFLAG and VpsT-3xFLAG run at the same size on the protein gel. Lanes 1-2 show RbmA-3xFLAG and lanes 3-4 show VpsT-3xFLAG. Bottom: Quantitation of VpsT-3xFLAG, RbmA-3xFLAG, and VpsR-3xFLAG protein levels from western blots performed on the indicated strains with the indicated treatments, as in the top panel. The AI-2-responsive strain is designated Control. Data are normalized to protein levels in the AI-2 treatment condition

(left set of bars). Unpaired *t*-tests were performed for statistical analyses. $^{**}P \le 0.01$; $^{*}P \le 0.05$; ns $P > 0.05$. Norspermidine, Nspd.

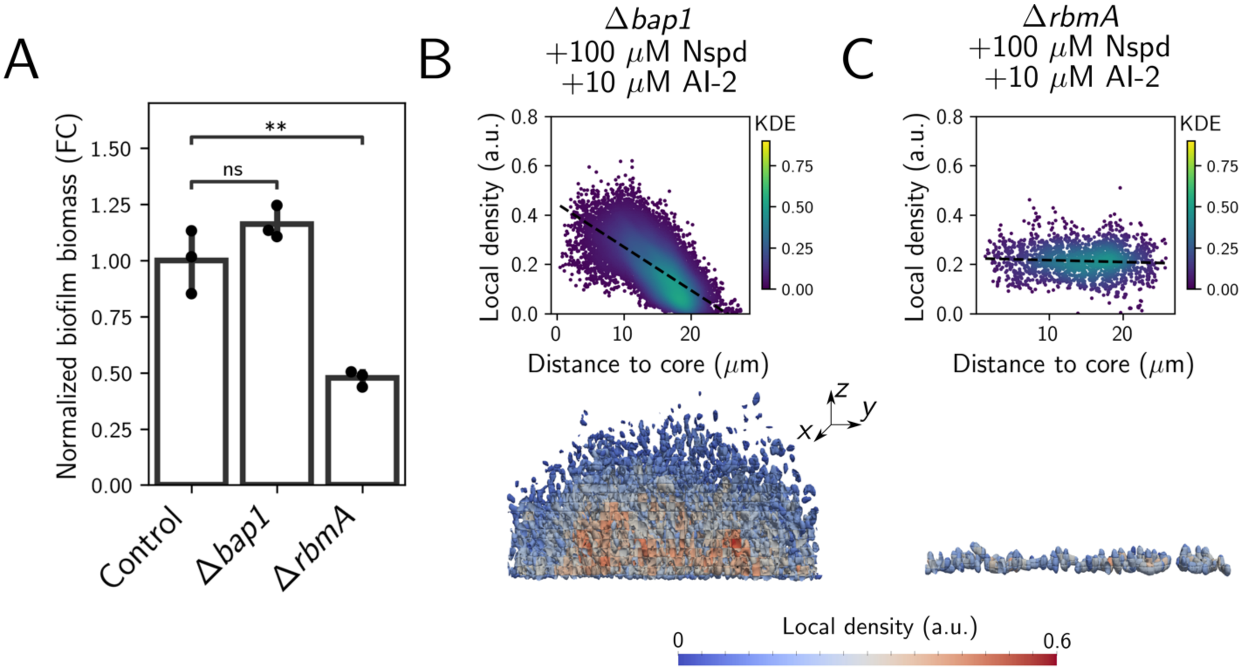


Fig. S5. **Activation of *rbmA* expression but not *bap1* expression drives changes in biofilm formation in the high cell density and high norspermidine signaling regime.** (A) Peak biofilm biomass, measured by quantitative brightfield imaging of biofilms produced by the AI-2-responsive strain (designated Control), the Δ*bap1* AI-2-responsive strain, and the Δ*rbmA* AI-2-responsive strain, each treated with both norspermidine and AI-2. (B-C) (Top panels) Scatter plots showing the relationship between local biofilm cell density and distance from the biofilm core. (Bottom panels) Cross-sectional 3D renderings of segmented cells in biofilms ~16 h post-inoculation, colored by local biofilm cell density. (B) In the Δ*bap1* AI-2-responsive strain treated with norspermidine and AI-2. (C) In the Δ*rbmA* AI-2-responsive strain treated with norspermidine and AI-2. In A, unpaired *t*-tests were performed for statistical analyses. $^{**}P \le 0.01$; ns $P > 0.05$. Norspermidine, Nspd; Kernel density estimate, KDE.

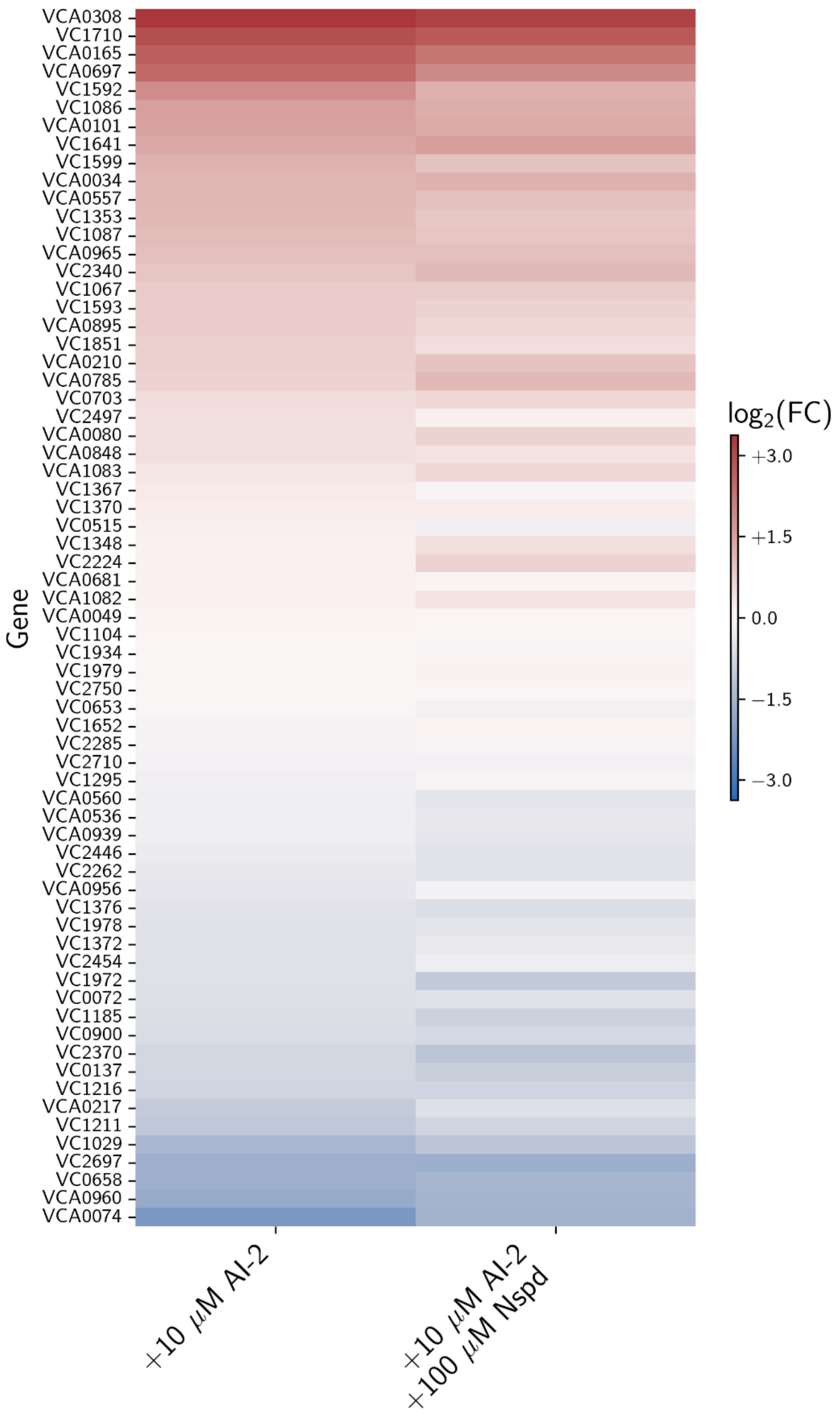


Fig. S6. **Transcriptional regulation of genes encoding c-di-GMP metabolizing enzymes by the high cell density quorum-sensing state.** Heatmap of $\log_2$ fold changes in the expression of genes encoding diguanylate cyclases and phosphodiesterases in the AI-2-responsive strain

grown with the indicated treatments normalized to transcript levels in the untreated strain. Samples were collected at $OD_{600}$ = 0.1. Norspermidine, Nspd; Fold change, FC.

Table S1: DNA oligonucleotides and gene fragments used in this study

| Oligo # | Name | Purpose | Direction | 5' to 3' Sequence |
|---|---|---|---|---|
| 113 | *hapR*_3000up | Cloning at *hapR* locus | F | CAGTGGCACATCATCGTCATC |
| 114 | *hapR*_3000down | Cloning at *hapR* locus | R | CACGCTGAACCACACATTGTTC |
| 262 | *hapR*_100up | Cloning at *hapR* locus | F | CACCCAACAGAGATTGACCTTG |
| 263 | *hapR*_100down | Cloning at *hapR* locus | R | GTGGGGATTCACTCATTTGCCG |
| 386 | *1807*_1500up | Cloning at *vc1807* locus | F | GCTCTAACCGTATATACAACTCCAAGTGG |
| 387 | *1807*_1500down | Cloning at *vc1807* locus | R | CCGTTTCATGCCCTACTCGCTAAC |
| 571 | *mbaA*_3000up | Cloning at *mbaA* locus | F | GCGCGCTAATCTGAACTCAACCCATAAG |
| 572 | *mbaA*_2700down | Cloning at *mbaA* locus | R | CGTTAGCATTCCACGCGGTCAGTTAG |
| 691 | *nspS*_3000up | Endogenous *Pbad-nspS-mbaA-3xFLAG* | F | GACTTTATCAGGCCTACTCGCGTTATCCCTG |
| 1073 | *Pbad-nspS*_ endogenous_B | Endogenous *Pbad-nspS-mbaA-3xFLAG* | R | GAATGATGTAGCCGTCAAGTTGTCATAA GATCCGACAAATAACCTAATAGCGTAAAAG |
| 1074 | *Pbad-nspS*_ endogenous_C | Endogenous *Pbad-nspS-mbaA-3xFLAG* | F | CTTTTACGCTATTAGGTTATTTGTCGGATC TTATGACAACTTGACGGCTACATCATTC |
| 985 | *Pbad-nspS*_B | Endogenous *Pbad-nspS-mbaA-3xFLAG* | R | CGATACCCATTCGTTACAAAAATTGGT CATTTCACACCTCCTGCAGGTAC |
| 892 | *Pbad*_*1807* *nspS*_C | Endogenous *Pbad-nspS-mbaA-3xFLAG* | F | GTACCTGCAGGAGGTGTGAAATG ACCAATTTTTGTAACGAATGGGTATCG |
| 674 | *mbaA*_SNAP_R | Endogenous *Pbad-nspS-mbaA-3xFLAG* | R | GAAGCCATGGGGAGATCTCGCTTAGGCTC |
| 692 | *nspS*_2700up | Endogenous *Pbad-nspS-mbaA-3xFLAG* | F | CTCTACAAAGCGGAACGTGGTTAACCG |
| 696 | *nspS*_100dwn | Endogenous *Pbad-nspS-mbaA-3xFLAG* | R | GATGATGTCACTTTTGGCCAGCGTG |
| 693 | *nspS*_100up | Endogenous *Pbad-nspS-mbaA-3xFLAG* | F | CTACCCTAGTGAAAACGTCAAACCCACTCAG |
| 914 | *3xFLAG-nspS*_B | Endogenous *Pbad-nspS-mbaA-3xFLAG* | R | CGCTGGCTTGATAACAGACAGTATTC |
| 1104 | *vpsR*_3000up | Cloning at *vpsR* locus | F | TTTTTCAGTAGAATTCCGCGGTTA |
| 1105 | *vpsR*_3000down | Cloning at *vpsR* locus | R | GTCGAGAGAAGCATCAATCCAATCT |
| 1106 | *vpsR*_2700up | Cloning at *vpsR* locus | F | TCGAAGGTCTACTAGAACTGGCCA |
| 1107 | *vpsR*_2700down | Cloning at *vpsR* locus | R | CGATGAAGCAGGCATGACCGTA |
| 1118 | *vpsR*_100up | Cloning at *vpsR* locus | F | CAGATTCTAGTGCAAAGTCTCGCT |
| 1119 | *vpsR*_100down | Cloning at *vpsR* locus | R | GCCATAACACTGAAATTCGCTGTA |
| 359 | *Pbad*_100up | Cloning *Pbad* promoter | F | GTCCACATTGATTATTTGCACGGCG |
| 741 | *3xFLAG*_Rev | Cloning *3xFLAG* | R | TCACTTGTCATCGTCATCCTTGTAATCG |
| 1100 | *vpsT*_3000up | Cloning at *vpsT* locus | F | AAGAAGTACCTCAACATTCGCACG |

| | | | | |
|---|---|---|---|---|
| 1141 | *vpsT*_3000down_correct | Cloning at *vpsT* locus | R | CATGACCATGGCGGACAAAATT |
| 1102 | *vpsT*_2700up | Cloning at *vpsT* locus | F | TACTGTAGAAACGCTGCAAATTTGT |
| 1103 | *vpsT*_2700down | Cloning at *vpsT* locus | R | AAAATTGAAGGCAAAGTAATGATCG |
| 1116 | *vpsT*_100up | Cloning at *vpsT* locus | F | CAGATTCCGCTTGATTAAACGTTT |
| 1140 | *vpsT*_100down_correct | Cloning at *vpsT* locus | R | TGTTTCAGGTACTCAGAAGGTAATT |
| 1150 | *bap1*_3000up | Cloning at *bap1* locus | F | AATCAAACCGGGCTTTAAATTTCA |
| 1155 | *bap1*_3000down | Cloning at *bap1* locus | R | CATCTACTGAAAGAGGTGCATAAG |
| 1152 | *bap1*_100up | Cloning at *bap1* locus | F | TCAGCATATCGTTATTGATGCCA |
| 1153 | *bap1*_100down | Cloning at *bap1* locus | R | ACTCTTGATTATAAGCAACGAAGTA |
| 1142 | *rbmA*_3000up | Cloning at *rbmA* locus | F | GCCAATTGTTGTACTGCTTTATGC |
| 1147 | *rbmA*_3000down | Cloning at *rbmA* locus | R | AAACCGCATCGTTCGCTTGATA |
| 1144 | *rbmA*_100up | Cloning at *rbmA* locus | F | TTAATGCTTAGCCAATGCAATTGT |
| 1145 | *rbmA*_100down | Cloning at *rbmA* locus | R | CCCTAAAATGCCTCATCACATCAT |
| 1087 | GSlinker_forward | $\Delta$*vc1807*::*Pbad-rbmA-3xFLAG* | F | GGTTCAGGAAGTGGTAGTGGATCT |
| 106 | BBC1882 | $\Delta$*vc1807*::*Pbad-rbmA-3xFLAG* | R | CAATTTTGCTTTTGGACCATCCC |
| 270 | *1807*_2700up | $\Delta$*vc1807*::*Pbad-rbmA-3xFLAG* | F | GGCCGGCACTTTGATTACAATC |
| 1158 | *Pbad*_*1807*_Univ_B_2 | $\Delta$*vc1807*::*Pbad-rbmA-3xFLAG* | R | CAATTCACACCTCCTGCAGGTAC |
| 1148 | *Pbad-rbmA*_forward | $\Delta$*vc1807*::*Pbad-rbmA-3xFLAG* | F | TACCTGCAGGAGGTGTGAA<br>TTGTCTAACTTTAAAGGATCTAT |
| 1149 | *Pbad-rbmA*_reverse | $\Delta$*vc1807*::*Pbad-rbmA-3xFLAG* | R | AGATCCACTACCACTTCCTGA<br>ACCTTTTTTTACCACTGTCATTGACT |

| | | | | |
|---|---|---|---|---|
| 192 | *vpsT-3xFLAG* | Western blot for VpsT-3xFLAG | - | ATGAAAGATGAAAACAAA<br>CTAAACGTTAGAATGCTTTC<br>TGATGTTTGCATGCAATCCAGATT<br>GTTGAAAGAGGCGTTAGAATC<br>AAAACTTCCTTTGGCGCTGG<br>AAATTACACCATTTTCTGAGCTCT<br>GGCTTGAAGAGAATAAACCAGAAAGTC<br>GCAGTATTCAGATGCTGGTGATTGATT<br>ATTCTAGAATTTCTGATGATGTTTT<br>GACCGATTACAGCTCGTTTAAG<br>CACATCAGTTGTCCTGATGCGAA<br>AGAGGTCATCATAAACTGT<br>CCGCAGGATATTGAGCATAAG<br>CTGCTCTTTAAGTGGAATAATTTG<br>GCTGGAGTATTTTATATTGATGATGATAT<br>GGATACCCTGATCAAAGGCATGAG<br>TAAAATTTTGCAAGATGAAATGT<br>GGTTAACGCGTAAACTGGCC<br>CAAGAATACATTCTCCAT<br>TATCGTGCCGGTAACTCA<br>GTCGTGACCTCACAAATGTAC<br>GCAAAATTAACCAAAAGAGA<br>ACAACAGATTATCAAGTTACTTGGTA<br>GTGGTGCTTCTAATATTGAAATTGC<br>AGATAAACTCTTTGTGAGTGAAA<br>ATACAGTAAAAACACATCTGC<br>ATAATGTCTTTAAGAAAATTA<br>ATGCCAAAAATCGCTTGCAGG<br>CACTGATTTGGGCGAAAAATAATATT<br>GGAATTGAGGAAGTCAATTCTGG<br>TTCAGGAAGTGGTAGTGGAT<br>CTGACTACAAAGACCATGACGG<br>TGATTATAAAGATCATGATA<br>TCGATTACAAGGATGA<br>CGATGACAAGTGA |

| | | | | |
|---|---|---|---|---|
| 193 | *vpsR-3xFLAG* | Western blot for VpsR-3xFLAG | - | ATGAGCACTCAATTCCGTATGGATTCAGTA<br>CCTGGCTCTCTTGTTGTGGTGGGAGGTACC<br>TATGAACCCTGGCTGGCTGTGTTGGAAAAAG<br>TGGGTTGGCGCTGTACTCAAGTAGCAGAT<br>TTGCGCAAAGCCGATGCGTTATTTGTC<br>GAGACTGGGCCATGTATTGGTATT<br>GTGGATTTAAGCCATGATGAATTTA<br>GCCTTAACGGGATTGCGAATTTGGTGA<br>GTAGCCATAAGCAAGTCCGTTGGCTGGCG<br>TTCATTCGTGAGGCGCAGCTCAGTTCAGAT<br>ACCATTTGCCAATTTATCGTTAACTTCTG<br>TATCGACTTTTTTACTGCGCCGATCCCTGAT<br>GCTCAGTTGTTGAGCACCATTGGTCACCAAC<br>TTGGCATGCTGAAGTTGGAAAAG<br>AAAGTTTGGCCACATTTTGGCTCGGCCG<br>GCAACATGGGCTTGATTGGGGAATCTA<br>TGCCTATGAAGCGTTTGCGCGATCAGATCAAAC<br>GCATTGGCCCGACCGATGTCAGTAT<br>TTTGATCTATGGCGAAAGTGGTACTGGGAA<br>AGAAACGGTGGCTAAGGCGATTCACA<br>AAACATCGTCGCGTGCACAAAAACCT<br>TTCATTTCAGTTAATTGCCGAGCTAT<br>GTCAGAAAAGCGCCTTGAAAGCGAGCTGTTTGG<br>TCTTGGCGAAACGGAGGAAGGCCAACAAC<br>CCTTTTTGCTGCAAGCGGATGGT<br>GGCACACTGCTGCTAAACGATATTTT<br>GACCTTGCCTAAAAGCCAACAGTTGAA<br>CCTACTGCGTTTCTTACAAGAAGGGA<br>CTGTGGAGACGCGCCAAGGGGT<br>TCGTGCAGTCGATGTGCGTATT<br>CTGGCCGCCAATTCGTCAGA<br>CATCGAAAAAGCGCTGATCGAT<br>GGTGATTTTAACGAAGAGTTG<br>TACCACTACATTAACGTA<br>TTGCGGATTAATGTGCCTAGTT<br>TGAAAGAGCGCGCATCGGA<br>TATCGTGCTACTTGCTAAAC<br>ACTTTTTGCAAGAATAC<br>TCCAAAGAGTACAACGCCCA<br>GGCGCGTAGCTTCTCTGAT<br>GATGCCGTACGTGGGTTAAC<br>TCGCTACCATTGGCCGGGTAATGT<br>GCGTGAGCTGATGAACC<br>AGATCAAGCGTGTGGTATTA<br>ATGTCAGATACTGTGGTGCT<br>GGATGAGTCTCAGCTCGATCTT<br>CCCAAGCGCAGTGATGGCC<br>GTCGCAGCCTGAAAAGC<br>ATTCGGGAACGCTCCGAGCGTG<br>ATGCACTGCTGCTGGTGTT<br>GGAGTCGCACTCTGGGCAAGTT<br>TCAACGGCAGCCAAAGAGCTG<br>GGAGTATCGCGTGCAA<br>CCATGTATCGCTTACTGAA<br>TAAACACAACTTGATCACCGA<br>TGAAAACTTCGGTTCAG<br>GAAGTGGTAGTGGATCTG<br>ACTACAAAGACCATGACGGT<br>GATTATAAAGATCATGATA<br>TCGATTACAAGGATG<br>ACGATGACAAGTGA |

Table S2: Strains used in this study

| Strain Number | Genotype | Plasmid | Antibiotic Resistance | Origin |
|---|---|---|---|---|
| MJ_142 | Δ*cqsS* Δ*vpsS* Δ*cqsR* Δ*luxS* (AI-2-responsive strain) | - | - | Jemielita et al. 2018 |
| AB_Vc_499 | Δ*cqsS* Δ*vpsS* Δ*cqsR* Δ*luxS* Δ*vc1807*::$Kan^R$ | - | Kan | Bridges et al. 2019 |
| AB_Vc_505 | Δ*cqsA* Δ*vpsS* Δ*cqsR* Δ*luxS* Δ*vc1807*::$Kan^R$ | - | Kan | Bridges et al. 2019 |
| JP_Vc_1253 | Δ*mbaA* Δ*cqsS* Δ*vpsS* Δ*cqsR* Δ*luxS* Δ*vc1807*::$Kan^R$ Δ*lacIZ*::$Spec^R$ | - | Kan, Spec | NT of AB_Vc_499 |
| MJ_093 | Δ*luxQ* Δ*vpsS* Δ*cqsR* Δ*cqsA* (CAI-1-responsive strain) | - | - | Jemielita et al. 2018 |
| JP_Vc_1219 | Δ*cqsS* Δ*vpsS* Δ*cqsR* Δ*luxS* Δ*vc1807*::$Kan^R$ | pFY4357::$Gm^R$ | Kan, Gm | Conj of AB_Vc_499 |
| JP_Vc_1300 | *luxO*$^{D61E}$ Δ*hapR* Δ*vpsL* | pFY4357::$Gm^R$ | Gm | Conj of BB_Vc_0223 |
| JP_Vc_1299 | *luxO*$^{D61A}$ Δ*hapR* | pFY4357::$Gm^R$ | Gm | Conj of BB_Vc_0219 |
| JP_Vc_1303 | *luxO*$^{D61E}$ Δ*hapR* Δ*aphA* Δ*vpsL* | pFY4357::$Gm^R$ | Gm | Conj of BB_Vc_0226 |
| JP_Vc_1302 | *luxO*$^{D61A}$ Δ*hapR* Δ*aphA* Δ*vpsL* | pFY4357::$Gm^R$ | Gm | Conj of BB_Vc_0227 |
| JP_Vc_1249 | *luxO*$^{D61E}$ Δ*hapR* Δ*aphA* Δ*qrr*1-4 | pFY4357::$Gm^R$ | Gm | Conj of YS_087 |
| JP_Vc_1261 | Δ*hapR* Δ*vpsL* Δ*cqsS* Δ*vpsS* Δ*cqsR* Δ*luxS* Δ*vc1807*::$Kan^R$ Δ*lacIZ*::$Spec^R$ | pFY4357::$Gm^R$ | Kan, Spec, Gm | Conj of JP_Vc_1260 |
| JP_Vc_1261 | *mbaA-3xFLAG* Δ*cqsS* Δ*vpsS* Δ*cqsR* Δ*luxS* Δ*vc1807*::$Kan^R$ Δ*lacIZ*::$Spec^R$ | - | Kan, Spec | NT of AB_Vc_499 |
| JP_Vc_1319 | Δ*hapR* *mbaA-3xFLAG* Δ*cqsS* Δ*vpsS* Δ*cqsR* Δ*luxS* Δ*vc1807*::$Kan^R$ Δ*lacIZ*::$Cm^R$ | - | Kan, Cm | NT of JP_Vc_1270 |
| AB_Vc_1324 | *Pbad-nspS-mbaA-3xFLAG* Δ*cqsS* Δ*vpsS* Δ*cqsR* Δ*luxS* Δ*vc1807*::$Cm^R$ Δ*lacIZ*::$Spec^R$ | - | Spec, Cm | NT of JP_Vc_1270 |
| JP_Vc_1391 | *Pbad-nspS-mbaA-3xFLAG* Δ*cqsS* Δ*vpsS* Δ*cqsR* Δ*luxS* Δ*vc1807*::$Cm^R$ Δ*lacIZ*::$Spec^R$ | pFY4357::$Gm^R$ | Spec, Cm, Gm | Conj of AB_Vc_1324 |
| JP_Vc_1258 | Δ*cqsS* Δ*vpsS* Δ*cqsR* Δ*luxS* Δ*vc1807-Pbad-vpvC*$^{W240R}$::$Kan^R$ | - | Kan | Conj of MJ_142 |
| JP_Vc_1340 | Δ*vpsT* *vpsR-3xFLAG* *bap1-3xFLAG* Δ*cqsS* Δ*vpsS* Δ*cqsR* Δ*luxS* Δ*vc1807-Pbad-vpsT-3xFLAG*::$Kan^R$ Δ*lacIZ*::$Spec^R$ | - | Kan, Spec | NT of JP_Vc_1338 |
| JP_Vc_1343 | Δ*vpsT* *rbmA-3xFLAG* Δ*cqsS* Δ*vpsS* Δ*cqsR* Δ*luxS* Δ*vc1807-Pbad-vpsT-3xFLAG*::$Kan^R$ | - | Kan | NT of MJ_142 |
| JP_Vc_1387 | Δ*vpsR* *rbmA-3xFLAG* Δ*cqsS* Δ*vpsS* Δ*cqsR* Δ*luxS* Δ*vc1807*::$Cm^R$ | - | Cm | NT of JP_Vc_1347 |
| JP_Vc_1394 | Δ*vpsR* *vpsT-3xFLAG* Δ*cqsS* Δ*vpsS* Δ*cqsR* Δ*luxS* Δ*vc1807*::$Kan^R$ | - | Kan | NT of JP_Vc_1329 |
| JP_Vc_1339 | Δ*bap1* Δ*cqsS* Δ*vpsS* Δ*cqsR* Δ*luxS* Δ*vc1807*::$Cm^R$ | - | Cm | NT of MJ_142 |
| JP_Vc_1347 | *rbmA-3xFLAG* Δ*cqsS* Δ*vpsS* Δ*cqsR* Δ*luxS* Δ*vc1807*::$Kan^R$ | - | Kan | NT of MJ_142 |
| JP_Vc_1349 | Δ*rbmA* Δ*cqsS* Δ*vpsS* Δ*cqsR* Δ*luxS* Δ*vc1807-Pbad-rbmA-3xFLAG*::$Kan^R$ | - | Kan | NT of JP_Vc_1342 |

NT = Natural Transformation

Conj = Conjugation
